# The complete chloroplast genome of *Mussaenda pubescens* and phylogenetic analysis

**DOI:** 10.1038/s41598-024-55010-y

**Published:** 2024-04-21

**Authors:** Caibi Zhou, Fang Tao, Rupiao Long, Xiaoting Yang, Xingli Wu, Lan Xiang, Xiaolu Zhou, Teerayoot Girdthai

**Affiliations:** 1 School of Crop Production Technology, Institute of Agricultural Technology, Suranaree University of Technology, Nakhon Ratchasima, 30000 Thailand; 2 College of Biological Science and Agriculture, Qiannan Normal University for Nationalities, Duyun, 558000 China; 3 College of Horticulture, Fujian Agriculture and Forestry University, Fuzhou, 350002 China

**Keywords:** Morphological characteristics, *Mussaenda pubescens*, Chloroplast genomes, Phylogenetic relationship, Evolution, Genetics, Plant sciences

## Abstract

The chloroplast (cp) genome sequence of *Mussaenda pubescens*, a promising resource that is used as a traditional medicine and drink, is important for understanding the phylogenetic relationships among the *Mussaenda* family and genetic improvement and reservation. This research represented the first comprehensive description of the morphological characteristics of *M. pubescens*, as well as an analysis of the complete cp genome and phylogenetic relationship. The results indicated a close relationship between *M. pubescens* and *M. hirsutula* based on the morphological characteristics of the flower and leaves. The cp was sequenced using the Illumina NovaSeq 6000 platform. The results indicated the cp genome of *M. pubescens* spanned a total length of 155,122 bp, including a pair of inverted repeats (IRA and IRB) with a length of 25,871 bp for each region, as well as a large single-copy (LSC) region and a small single-copy (SSC) region with lengths of 85,370 bp and 18,010 bp, respectively. The results of phylogenetic analyses demonstrated that species within the same genus displayed a tendency to group closely together. It was suggested that *Antirhea*, *Cinchona*, *Mitragyna*, *Neolamarckia*, and *Uncaria* might have experienced an early divergence. Furthermore, *M. hirsutula* showed a close genetic connection to *M. pubescens*, with the two species having partially overlapping distributions in China. This study presents crucial findings regarding the identification, evolution, and phylogenetic research on *Mussaenda* plants, specifically targeting *M. pubescens*.

## Introduction

The *Mussaenda* genus contributes significantly to medical natural products, including flavonoids, triterpenes, saponins, and iridoids identified primarily from *M. pubescens*. The stems and leaves of *M. pubescens* with a sweet flavor possess the therapeutic effects of cooling and relieving summer heat, clearing heat, and dispersing wind, commonly used medicinally or dried as a tea substitute^1^. The whole plant of *M. pubescens* has been used in Chinese folk medicine against laryngopharyngitis, acute gastroenteritis, and dysentery^2^. Its extracts exhibit immunostimulant, hemolytic^3^, detoxifying^4^, analgesic^5^, and anti-RSV activities^6^. The genus *Mussaenda* L., which belongs to the family Rubiaceae belonging to *Rubiaceae*, consists of approximately 120 species primarily found in Africa, the Pacific islands, and Asia, including 31 species, 1 variety, and 1 variant in China. The morphology of trichomes and the shape of calyx and corolla are valuable characteristics for species identification^7^. The species of *Mussaenda* can be easily distinguishable from other genera due to their expanded petaloid calycophylls, terminal umbel inflorescence, and fleshy berries. *M. pubescens*, a liana-like shrub belonging to the genus *Musaenda*^8^, is categorized into two forms known as *M. pubescens* f. *pubescens* and *M. pubescens* f. *clematidiflora*, which are identified as the same species through ‘**the Key to Dioecious Species of *Musaenda* in China**’.

In general, the chloroplast is a semi-autonomous replication organelle^9^ and contains independent cpDNA^10^. Some species exhibit a circular structure consisting of one short single-copy (SSC) region, one large single-copy (LSC) region, and two inverted repeats (IR) regions that serve as a separator between the LSC and SSC regions^9^. The contraction and expansion of the IR, LSC, and SSC regions are common phenomena in the process of evolution^11^, which regions constitute the primary factors that contribute to the differences in size observed in cp genomes^12^. SSRs identified in cp genomes exhibit high conservation in terms of types and numbers, and the majority of mono-nucleotide repeats consist of base A or T, making them particularly well-suited as genetic markers for plant molecular studies^13^. The cp genome offers valuable insights for various scientific fields such as species identification, population genetics, phylogenetics, and genetic engineering research due to its similar structure^14,15^, large genetic information, highly conserved sequences^16^, high-valuable information^17^, and stable maternal inheritance^18^. Therefore, the cp genome has become an ideal resource for species and higher-order phylogenetic inference^19^, serving as a molecular marker in taxonomic research^20^. The complete chloroplast genomes of various plant species within the same genus were sequenced using the Illumina NovaSeq 6000 platform, thus providing valuable insights for species identification, evolutionary analysis, and phylogenetic studies of these plants^21^.

In the present study, the complete chloroplast genomes of *M. pubescens* were sequenced using Illumina technology and subsequently characterized their features. Our research aimed to investigate the molecular structures of *M. pubescens* cp genomes and analyze the phylogenetic relationships among *Mussaenda* species by utilizing their complete cp genome sequences, including the LSC, SSC, and IR regions. The current research will serve as a valuable resource for future studies on species identification, evolution, population genetics, and phylogenetic analyses of *Mussaenda*.

## Material and methods

### Plant material and morphological observations

**Figure 1:**
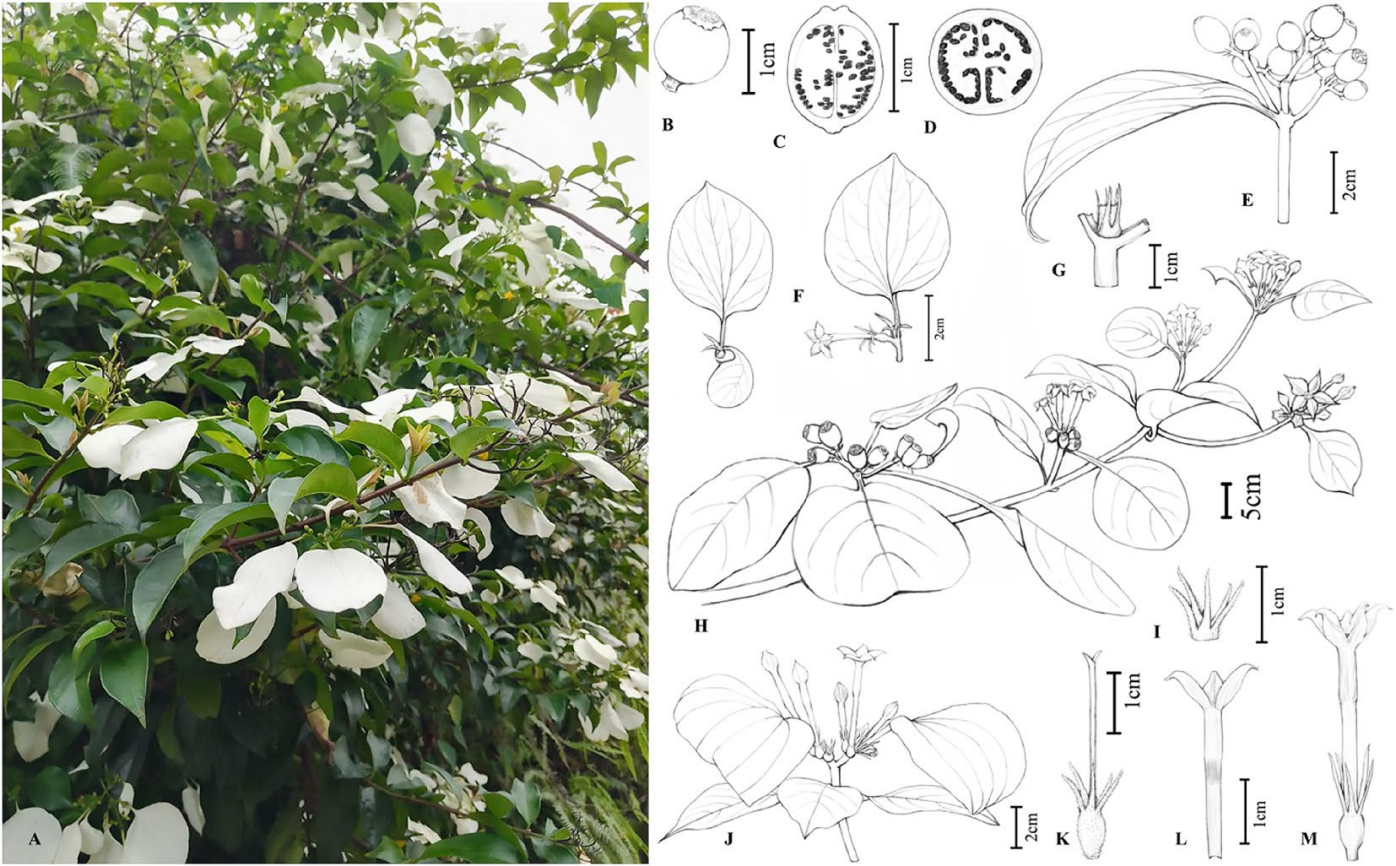
Morphological characters of *M. pubescens*. (**A**) Growth environment; (**B**) Fruit; (**C**) The longitudinal section of the fruit; (**D**) Transection of the fruit; (**E**) A fruiting branch (2 cm); (**F**) Enlarged Calyxlobes (2 cm); (**G**) Stipule (1 cm); (**H**) A long-styly branch (Scale bar: 5 cm); (**I**) Calyxlobes (1 cm); (**J**) A flowering short-styly branch (2 cm); (**K**) long-styly stigma (1 cm); (**L**) Long-styly corolla (1 cm); (**M**) Long-styly flower (1 cm).

All the materials were collected from the Third Botanical Resources Survey in Libo County (Guizhou, China, 26° 12′ N, 107° 30′ E) in September 2020. The species has been officially identified as ‘*M. pubescens*’ by a professor of plant taxonomy at Qiannan Normal University for Nationalities, which would be compared with the specimens of Chinese *Mussaenda*. The specimen (DZ20210505) was deposited in the herbarium of Qiannan Normal University for Nationalities in Duyun City, Guizhou Province, China. Morphological features of *M. pubescens* involved in branches, leaves, stipules, inflorescences, calyx lobes, corolla tubes, florescences, and fruits were observed to compare their differences (Fig. 1).

### Complete chloroplast genome sequence of * M. pubescens*

The leaves of *M. pubescens* were collected on September 15, 2020 (Fig. 1) and stored immediately at  − 80 °C. The isolation of total genomic DNA was performed using a modified CTAB method^22^. Library construction and sequencing were conducted by Novogene Bioinformatics Technology Co. Ltd. (Guangzhou, China) using the Illumina NovaSeq 6000 platform with a Paired-End 150 (PE150) strategy. The FastQC software was employed to trim low-quality reads and adapters, and the genome was de novo assembled using SPAdes v3.9^23^, and subsequently annotated using Plann software^22^. The accuracy of the preliminary annotation results was verified by comparing them with the proteins and rRNA sequences from previously reported cp genomes of relevant species using the methods of blastn and blastp. The complete chloroplast genome was submitted to the Database Resources of the National Genomics Data Center, China National Center for Bioinformatio (Genome Sequence Archive accession number: CRA013306; https://ngdc.cncb.ac.cn/gsa). The circular structure of the cp genome was visualized using CPGView (http://www.1kmpg.cn/cpgview/).

### Phylogenetic analysis

A total of 28 complete chloroplast genome sequences spanning across 24 Rubiaceae species were acquired from the NCBI GenBank to ascertain the phylogenetic positions of *M. pubescens* within the Rubiaceae lineages. The Bayesian inference (BI) tree was constructed following standard protocols. The alignment of 28 cp genomic sequences was generated using the MAFFT online version^24^ with default parameters. The software tools MAFFT, Ultrafast bootstrap, IQ-TREE, ModelFinder, and MrBayes were employed within the PhyloSuite framework^25^. FigTree was employed to illustrate the phylogenetic relationships (http://tree.bio.ed.ac.uk/software/figtree/).

### Ethics approval and consent to participate

*Mussaenda pubescens* is not endangered in China, and no specific permission was required for the collection. All Mussaenda pubescens materials in this study were collected in germplasm resource nursery of the College of Biological Science and Agriculture, Qiannan Normal University for Nationalities (QNUN) with the permission of the school. The current study complied with relevant institutional, national, and international guidelines and legislation.

## Results and discussion

### Morphological characteristics of * M. pubescens*

The collected specimens of *M. pubescens* were compared with Chinese *Mussaenda* specimens for this research study (Table 1). The morphological features of *M. pubescens*, including branches, leaves, stipules, inflorescences, calyx lobes, corolla tubes, florescences, and fruits, were presented in Fig. 1.

**Table 1:** Morphological comparison of *M. pubescens* and *M. hirsutula.*

Taxa	*M. pubescens*	*M. hirsutula*
Habitat	Climbing shrub, often extensively twining	Climbing shrubs
Branch	Branchlets, appressed pubescence	Branchlets, densely ferruginous- or gray villosulous
Leaf	Opposite or rarely whorled; obvious petiolate (3–15 mm), densely strigose; membranous or thin papery, ovoid oblong, ovoid lanceolate, 5–8 × 2–5 cm; apex abruptly acuminate or acute, base cuneate; with strigose sparsely on adaxially, densely on abaxially and veins; tertiary venation reticulate, secondary veins 4–7 pairs	Opposite; petiole 3–5 mm, densely pilose; papery, elliptic or oblong, subovate, 7–13 × 2.5–4 cm, apex mucronate or acuminate, base cuneate, sparsely pilose on both sides, denser on below and veins; secondary veins 6–7 pairs, tertiary venation visible and reticulate
Stipules	Triangular, 5–7 mm, densely strigillose; deeply 2-lobed, segments subulate, 4–6 mm long	Triangular, 3–5 mm, densely pilose, deeply 2-lobed or 2-lobed; lobes lanceolate, 3–5 mm long
Inflorescences	Terminal, subcapitate, mostly unbranched, cyme, 4–6 × 2–7 cm, dense flowered, densely strigillose to villosulous; peduncles (sessile to nearly sessile, although fruits may have stalks) 0.1–1.4 cm; bracts, linear, 3–5 mm	Terminal, cymose, 1.5–4 × 1.5–4 cm, densely grayish yellow villosulous; peduncle 0.3–1.5 cm; bract linear lanceolate, 4–5 mm
Calyx	Calyx lobe, non-leaflike, linear or triangular, less than 1.5 mm wide, longer than corolla tube twice, densely pilose on base, becoming sparser upward; 1 lobes on 1–3 flowers per inflorescence expanded into calycophyll, broadly elliptic, 2.5–5 × 2–3.5 cm, blunt or mucronate on apex, narrow on base strigillose on both surfaces, with 5–7 longitudinal veins	Calyx lobe elliptic, 4–5 mm long, densely pilose; lobe, linear, 7–10 mm long, densely pilose; 1 lobe on 1–3 flowers per inflorescence expanded into calycophyll, broadly elliptic, 4–4.5 × 3–3.5 cm, round or short pointed on apex, nearly rounded on base, pilose, with 7 longitudinal veins
Corolla	Corolla yellow, outside pubescent; corolla tube gyro-shaped, 3–4 mm long, uniformly cylindrical or inflated just below or at throat and densely clavate pubescent; corolla lobes, 4 mm long, oblong-lanceolate, acuminate	Corolla yellow, outside densely strigillose; corolla tube, 26–28 mm, constricted at throat and inside orange-yellow clavate pubescent; corolla lobes, ellipsoid, mucronate
Flower period	April–July	April–June
Fruit	Fruit period, Jun to Dec; berry subglobose, 8–10 × 6–7.5 mm in diameter, smooth, fleshy or stiffly papery, sparsely pilose; stipitate 4–5 mm; black after drying	Fruit period, July to next January; berry ellipsoid or subglobose, 14–20 × 9–12 mm in diameter; brown after dry with small light brown spots
Identification	Inflorescence from congestion to relaxation Flowers with stalks 1–5 mm Corolla tube, gyro-shaped (3–4 mm long). Calyx lobes, non-leaflike, linear or triangular, less than 1.5 mm wide, 3–6 mm long, longer than corolla tube twice Leaves with distinctly petiolate, were ovate-oblong or ovate-lanceolate	Inflorescences densely congested Flowers sessile or subsessile Calyx lobes longer than corolla tube, twice Calyx lobes, linear; corolla tube, elliptic; corolla lobes, elliptic Leaves, elliptic or oblong, rarely obovate

**Type**: China. Guizhou, Libo County, Maolan National Nature Reserve, 22° 5′ N, 107° 56′ E, ca. 980 m a.s.l., on forest edge, 26 Sep 2020, S. Wang 161915 (Holotype, IBSC!).

**Distribution and habitat**: Currently, *M. pubescens* is known from the provinces of Zhejiang, Hainan, Yunnan, Guizhou, Sichuan, Guangxi, Guangdong, Hubei, Fujian, Hunan, Jiangxi, and Taiwan in China. This plant species is commonly found in dense thickets located in ravines, on slopes of hills, and along village boundaries or roadsides, typically thriving at elevations ranging from 100 to 900 m a.s.l.

**Phenology**: Flowering from April to July, and fruiting from June to November.

**Description**: *M. pubescens*, climbing shrub, often extensively twining. Branchlets appressed pubescence, the stems are often accompanied by axillary short shoots that bear small leaves. Leaves with distinctly petiolate (3–15 mm), were ovate-oblong or ovate-lanceolate (5–8 × 2–5 cm), opposite or perhaps rarely whorled, membranous or thinly papery, densely strigillose. The stipules were triangular, densely strigillose, deeply 2-lobed, segments subulate (4–6 mm long). Inflorescences were cyme with sessile to subsessile (0.1–1.4 cm, though the fruit may be stipitate), primarily unbranched, densely strigillose to villosulous. Calyx lobes were non-leaflike, linear or triangular, less than 1.5 mm wide, longer than corolla tube twice, densely pilose on base, becoming sparser upward; 1 lobe on 1–3 flowers per inflorescence expanded into calycophyll, broadly elliptic (2.5–5 × 2–3.5 cm), narrow on base, strigillose on both surfaces, with 5–7 longitudinal veins. Corolla tubes were gyro-shaped (3–4 mm long), uniformly cylindrical, or inflated just below or at the throat with dense clavate pubescence; corolla lobes were oblong-lanceolate (4 mm long) and acuminate. The flower period was Apr-Jul. Fruit period was Jun to Dec, were berry subglobose, 8–10 × 6–7.5 mm in diameter, smooth, fleshy or stiffly papery, sparsely pilose; stipitate 4–5 mm; black after drying.

**Similar species**: The breeding system of *M. pubescens* is characterized by functional dioecy. A **Key to Dioecious Species of *Mussaenda* in China** is provided below. The forms of trichomes, as well as the morphology of the calyx and corolla, are important characteristics for identifying species^7^. *M. pubescens* is easily distinguished by its corolla tube 11–20 mm, with stems frequently having axillary short shoots containing small leaves^26,27^, flowers sessile to pedicellate and range in color from white to yellow, despite bearing morphological similarities to *M. hirsutula*^28^. Differences among these two species are provided in Table 1.

**Etymolog**y: The specific epithet is derived from the shape of the corolla.

**Paratypes**: China, Guangxi: Liu Jun 1235399 (PPBC), Zhang Dianxiang 0153214 (IBSC), Deng Liang 0659417 (IBSC), Rong County Census Team 0205196 (GXM); Guangdong: Li Guangmin 149045 (PPBC), Deng Xiaotong 012602 (SN), Liu Na 012311 (SN), Yang Yuying 010881 (SN); Hunan: Yu Xunlin 1236160 (PPBC), Ran Jing 0007240 (GNUG); Yunnan: Chen Yousheng 449219 (PPBC), Li Yongliang 1450785 (KUN), Yu Xunlin 064698 (CSFI); Fujian: Qu Hua 068014 (AU), Ching H.H. 0659418 (IBSC), Guo Zhizhao 076512 (AU); Jiangxi: Cao Lan 0001459 (JXCM), Chen Gongxi 00197444 (SYS); Gui zhou: Li Qirui 0101386 (GZTM), Chen Jianxiang 0099290 (GZTM), Wu Shiyan 0102818 (GZTM); Taiwan: Masakazu 0768530 (IBSC), Bartholomew B. 00793088 (PE); Hong Kong: Shiu Ying 00793108 (PE), Chen Huanyong 00793121 (PE); Sichuan: Zhu Dahai 02115800 (PE), Fang Wenpei 0472099 (IBSC); Hubei: Fu Guoxun 0472100 (IBSC); Zhejiang: Qin Renchang 0472098 (IBSC).

### Key to dioecious species of * Mussaenda* in China

- 1a. Individual flowers with all calyx lobes enlarged into petaloid calycophylls……………*M. anomala*
- 1b. Individual flowers with only 1 calyx lobes enlarged into petaloid calycophylls, or without calycophylls………(2)
- 2a. Normal calyx lobes subleaflike, lanceolate, 1.5–5 mm wide…………………(3)
- 3a. Branches, calyx, and corolla, covered villosulous………………*M. macrophylla*
- 3b. Branches, calyx and corolla, appressed strigillose …………………*M. esquirolii*
- 2b. Normal calyx lobes, non-leaflike, linear or triangular, less than 1.5 mm wide……………(4)
- 4a. Leaves, sessile or subsessile, < 3 mm
- 4b. Leaves, distinctly petiolate, 3–15 mm………………………(5)
- 5a. Normal calyx lobes (at anthesis) 1–15 mm, shorter than calyx tube
- 5b. Normal calyx lobes (at anthesis) 25–30 mm, longer than calyx tube………………(6)
- 6a. Calyx lobes longer than calyx tube, but not more than twice………………(7)
- 7a. Cymes, dense
- 7b. Cymes, Loose…………………………(8)
- 8a. Calyx lobes, linear
- 8b. Calyx lobes, subulate………………………(9)
- 9a. Corolla lobes, triangular-ovate; leaves, elliptic………………*M. elliptica*
- 9b. Corolla lobes, ovate; leaves, elliptic or ovate-elliptic, with pubescent on abaxially……*M. divaricata*
- 6b. Calyx lobes longer than calyx tube, more than twice and more………………(10)
- 10a. Calyx lobes longer than calyx tube, more than twice
- 10b. Calyx lobes longer than corolla tube, twice …………………(11)
- 11a. Corolla tube, elliptic; leaves, elliptic or oblong, rarely obovate……………(12)
- 12a. Corolla lobes, triangular-ovate; calyx lobes, linear-lanceolate…………*M. hainanensis*
- 12b. Corolla lobes, elliptic; calyx lobes, linear ………………*M. hirsutula*
- 11b. Corolla tube, gyro-shaped, 3–4 mm long; leaves, ovate-oblong or ovate-lanceolate; corolla lobes, oblong-lanceolate, acuminate ……………………*M. pubescens*

### Chloroplast genome features of M. pubescens

Chloroplast DNA sequences, characterized by their highly conserved structure, minimal recombination events, and predominantly uniparental inheritance, have traditionally served as preferred markers for reconstructing plant phylogeny. A robust phylogeny is essential to investigate the evolutionary patterns of traits across varying taxonomic levels. The chloroplast genome yields essential information for various scientific disciplines, including species identification, population genetics, phylogenetics, and genetic engineering research^14,15,29^. The filtering process obtained 20,928,581 paired-end reads from the Illumina NovaSeq platform, all meeting the required quality standards. The values for Q20 and Q30 were determined to be 97.53% and 92.98%, respectively. The complete cp genome sequence of *M. pubescens* was constructed de novo and subsequently submitted to the Database Resources of the National Genomics Data Center (GSA accession number: CRA013306).

**Figure 2:**
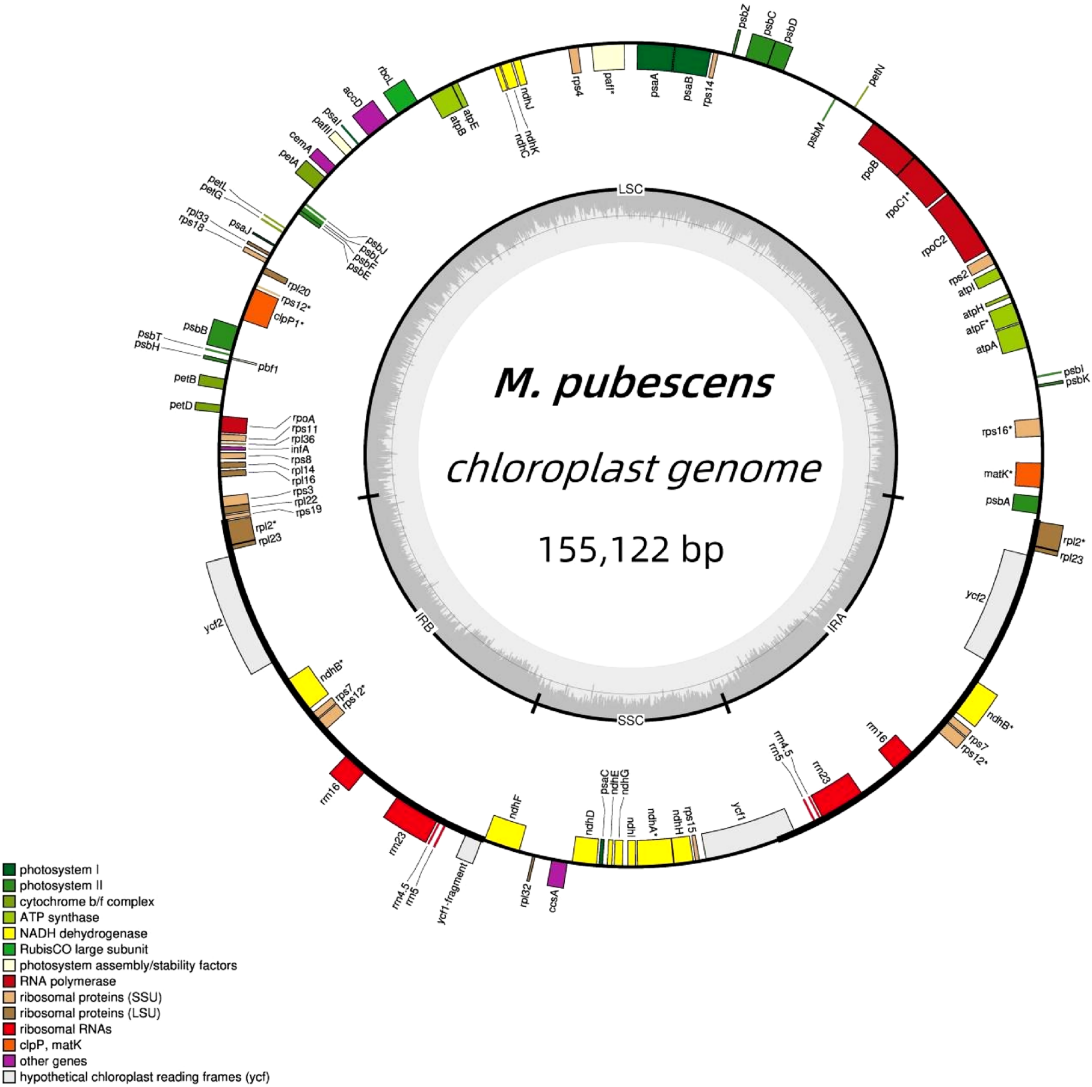
The chloroplast genome maps of *M. pubescens*. Genes situated on the inner side of the circle are transcribed in a clockwise direction, whereas those on the outer side are transcribed in a counter-clockwise direction. The inner circle of a darker gray represents the GC content, while the lighter gray indicates the AT content. Different colors represent different functional genes.

The chloroplast genomes of plants consisted of photosynthetic genes, genes related to chloroplast transcriptional expression, and additional protein-coding genes^30^. Expansion and contraction of the boundaries of the inverted region (IR) are the primary factors contributing to changes in the size of the cp genomes, playing a crucial role in species evolution^31^. As was common with other angiosperms, the cp genome of *M. pubescens* consisted of a circular genome measuring 155,122 bp in length and possessing the typical quadripartite structure. This structure included a pair of reverse repeats, namely IRA and IRB, which spanned 25,871 bp, a small single copy region (SSC) measuring 18,010 bp, and a large single copy region (LSC) spanning 85,370 bp (Table 2 and Fig. 2). The expansion and contraction observed in the IR regions might serve as the primary mechanism for generating variations in the length of the cp genomes in both *M. pubescens* and its closely related species^9,32^.

**Table 2:** Characteristics of *M. pubescens* cp genome.

Category	Item (bp)	Describe
cp genome structure	Total length	155122
	LSC length	85370
	SSC length	18010
	IRA length	25871
	IRB length	25871
Gene composition	Genes	128
	Protein-coding genes (CDS)	86
	tRNA genes	34
	rRNA genes	8

The GC content varied across different regions of the cp genomes, with the IR regions particularly exhibiting high GC content, likely due to the presence of rRNAs^33^. Our findings indicated that the GC contents in the IR and SSC regions were measured to be 43.17% and 31.89%, respectively, with the average GC content of the whole genome being 37.67% (Table 3). Notably, the GC content of DNA in the IR regions was higher than that in other regions (LSC, SSC) (Fig. 2), which aligned with similar patterns observed in other flowering plants^34,35^. Furthermore, the GC skewness has been identified as a crucial indicator of DNA leading chains, lagging chains, and replication origins and terminals, which in turn serves as an important determinant of species affinity^36^.

**Table 3:** The nucleotide composition of the complete chloroplast genomes of *M. pubescens.*

Region	A/%	T(U)/%	C/%	G/%	AT/%	GC/%
LSC	31.59	32.86	18.24	17.31	64.45	35.55
SSC	34.04	34.08	16.64	15.25	68.11	31.89
IR	28.42	28.42	21.58	21.58	56.83	43.17
Total	30.82	31.52	19.17	18.50	62.33	37.67

Gene functions were subsequently assigned to all the genes (Table 2), with these genes being classified into four types: genes related to self-replication, genes related to photosynthesis, unknown function genes, and specific genes, including maturase (matK), protease (clpP), and others. Out of the 128 identified genes, there were 86 protein-coding genes, 34 transfer RNA (tRNA) genes, and 8 rRNA genes. The observed results were similar to those found in other *Mussaenda* species^37,38^.

In total, 62 protein-coding genes and 22 tRNA genes were situated in the LSC region, while 11 protein-coding genes and 1 tRNA gene were assigned to the SSC region of the cp genome (Fig. 2). Additionally, a total of 20 intron-containing genes, among which there were 17 genes (*e.g.*, *ndhA*, *ndhB*, *petB*, *petD*, *atpF*, *rpl16*, *rpl2*, and *rps16*) with 1 intron and only 3 genes (*rps12*, *clpP1*, and *trnH-GUG*) with 2 introns (Table 4). Among them, *ndhA* possessed the longest intron (1115 bp), while the shortest intron (16 bp) was observed in *trnI GAU* (Table 5). It was noteworthy that *rps12* was classified as a trans-spliced gene consisting of two separated introns, with one exon positioned in the LSC region and the other two in the IR region (Fig. 2). Besides that, the *rps12* gene of *M. pubescens* was comprised of an exon at the 5’-end located in the LSC region, while its 3’-end exons were positioned in the IR regions, as depicted in Fig. 2, which was consistent with that of the homologous species *M. hirsutula*^38^.

**Table 4:** Lists of annotated genomic genes for *M. pubescens.*

Category	Gene group	Gene name
Photosynthesis	Subunits of photosystem I	*psaA*, *psaB*, *psaC*, *psaI*,* psaJ*
	Subunits of photosystem II	*psbA*, *psbB*, *psbC*, *psbD*, *psbE*, *psbF*, *psbH*, *psbI*, *psbJ*, *psbK*, *psbL*, *psbM*, *psbN*, *psbT*, *psbZ*
	Subunits of NADH dehydrogenase	ndhA*, ndhB* (2), *ndhC*, *ndhD*, *ndhE*, *ndhF*, *ndhG*, *ndhH*, *ndhI*, *ndhJ*, *ndhK*
	Subunits of cytochrome b/f complex	*petA*, petB*, petD*, *petG*, *petL*, *petN*
	Subunits of ATP synthase	*atpA*, *atpB* (2), *atpE*, atpF*, *atpH*, *atpI*
	Large subunit of rubisco	*rbcL*
	Subunits photochlorophyllide reductase	–
Self-replication	Large subunit ribosomal proteins	*rp114*, rp116*, rp12*(2), *rp120*, *rp122*,* rp123* (2), *rp132*, *rp133*, *rp136*
	Small subunit ribosomal proteins	*rps11*, rps12** (2), *rps14*, *rps15*, rps16*, *rps18*, *rps19*, *rps2*, *rps3*, *rps4*, *rps7* (2), *rps8*
	Subunits of RNA polymerase	*rpoA*, *rpoB*, rpoC1*, *rpoC2*
	Ribosomal RNAs	*rrn16* (2), *rrn23* (2), *rrn4.5* (2), *rrn5* (2)
	Transfer RNAs	trnA-UGC*(2), *trnC-GCA*, *trnD-GUC*, *trnE-UUC*, *trnF-GAA*, trnG-GCC*, trnH-GUG**(2), *trnH-GUG*, trnI-GAU* (2), trnL-CAA* (2), *trnL-UAG*, *trnM-CAU* (2), trnM-CAU* (2), trnN-GUU* (2), *trnP-UGG*, *trnQ-UUG*, trnR-ACG* (2), *trnR-UCU*, *trnS-GCU*, *trnS-GGA*, *trnS-UGA*, *trnT-GGU*, *trnT-UGU*, trnV-GAC* (2), *trnW-CCA*, *trnY-GUA*
Other genes	Maturase	*matK*
	Protease	clpP1**
	Envelope membrane protein	*cemA*
	Acetyl-CoA carboxylase	*accD*
	c-type cytochrome synthesis gene	*ccsA*
	Translation initiation factor	*infA*
	Other	–
Unknown function genes	Conserved hypothetical chloroplast ORF	*pafI*, *pafII*, *pbf1*, *ycf1*, *#ycf1*, *ycf2* (2)

*, Gene with one intron; **, Gene with two introns; #, Pseudogene; (2), Gene with two copies.

**Table 5:** Characteristics and sizes of the intron and exon genes from* M. pubescens.*

Gene	Region (bp)	Exon I (bp)	Intron I (bp)	Exon II (bp)	Intron II (bp)	Exon III (bp)
*rps16*	LSC	36	840	231		
*atpF*		144	715	411		
*rpoC1*		430	756	1613		
*pafI*		124	717	228	768	155
*clpP1*		71	789	292	663	228
*rpl2*	IRB	391	659	434		
*ndhB*		777	684	756		
*rps12*		114	–	234	526	25
*trnI-GAU*		36	16	36		
*trnA-UGC*		37	40	28		
*ndhA*	SSC	553	1115	539		
*trnA-UGC*	IRA	37	40	28		
*trnI-GAU*		36	16	36		
*rps12*		234	526	25		
*ndhB*		777	684	756		
*rpl2*		391	659	434		

The codon usage bias in chloroplast genomes may arise from a combination of natural selection and genetic mutation, which is important to investigate this phenomenon as it provides insights into the evolutionary processes and functional constraints shaping the genetic code of chloroplasts^39^. The relative frequency of synonymous codons in the coding sequence of *M. pubescens* cp demonstrated that all genes were represented by 19,850 codons. The study identified the four most commonly utilized codons as ATT (Isoleucine), GAA (Glutamic acid), AAT (Asparagine), and AAA (Lysine), accounting for 843 (4.25%), 752 (3.79%), 723 (3.64%), and 715 (3.60%) codons, respectively. One of the most commonly used amino acids was leucine, with 2155 hits; another one, cysteine, had the lowest content, with only 277 hits. Additionally, codons ending with ‘A’ and ‘T’ accounted for 69.13% of all codons (Table 6), which aligned with previous studies on angiosperms^40^. The codon usage preferences encompassed within these features could actively contribute to a more in-depth understanding of exogenous gene expression and the mechanisms driving the evolution of the cp genome^41,42^.

**Table 6:** Codon usage of *M. pubescens* cp genome from RSCU tools.

Amino acid	Symbol	Codon	Number	/1000	RSCU
*	Ter	TGA	13	0.65	0.57
*	Ter	TAG	21	1.06	0.93
*	Ter	TAA	34	1.71	1.50
A	Ala	GCG	100	5.04	0.37
A	Ala	GCA	301	15.16	1.11
A	Ala	GCT	480	24.18	1.77
A	Ala	GCC	201	10.13	0.74
C	Cys	TGT	176	8.87	1.55
C	Cys	TGC	51	2.57	0.45
D	Asp	GAT	652	32.85	1.62
D	Asp	GAC	153	7.71	0.38
E	Glu	GAG	267	13.45	0.52
E	Glu	GAA	752	37.88	1.48
F	Phe	TTT	672	33.85	1.23
F	Phe	TTC	420	21.16	0.77
G	Gly	GGG	252	12.7	0.72
G	Gly	GGA	580	29.22	1.66
G	Gly	GGT	437	22.02	1.25
G	Gly	GGC	127	6.4	0.36
H	His	CAT	380	19.14	1.53
H	His	CAC	118	5.94	0.47
I	Ile	ATA	511	25.74	0.91
I	Ile	ATT	843	42.47	1.50
I	Ile	ATC	335	16.88	0.60
K	Lys	AAG	270	13.6	0.55
K	Lys	AAA	715	36.02	1.45
L	Leu	TTG	431	21.71	1.20
L	Leu	TTA	618	31.13	1.72
L	Leu	CTG	140	7.05	0.39
L	Leu	CTA	329	16.57	0.92
L	Leu	CTT	479	24.13	1.33
L	Leu	CTC	158	7.96	0.44
M	Met	ATG	453	22.82	1.00
N	Asn	AAT	723	36.42	1.52
N	Asn	AAC	230	11.59	0.48
P	Pro	CCG	120	6.05	0.59
P	Pro	CCA	230	11.59	1.13
P	Pro	CCT	296	14.91	1.46
P	Pro	CCC	165	8.31	0.81
Q	Gln	CAG	189	9.52	0.52
Q	Gln	CAA	540	27.2	1.48
R	Arg	AGG	136	6.85	0.67
R	Arg	AGA	345	17.38	1.71
R	Arg	CGG	99	4.99	0.49
R	Arg	CGA	303	15.26	1.50
R	Arg	CGT	250	12.59	1.24
R	Arg	CGC	81	4.08	0.40
S	Ser	AGT	284	14.31	1.07
S	Ser	AGC	95	4.79	0.36
S	Ser	TCG	170	8.56	0.64
S	Ser	TCA	320	16.12	1.21
S	Ser	TCT	455	22.92	1.72
S	Ser	TCC	267	13.45	1.01
T	Thr	ACG	130	6.55	0.53
T	Thr	ACA	296	14.91	1.20
T	Thr	ACT	365	18.39	1.48
T	Thr	ACC	193	9.72	0.78
V	Val	GTG	156	7.86	0.59
V	Val	GTA	400	20.15	1.50
V	Val	GTT	373	18.79	1.40
V	Val	GTC	136	6.85	0.51
W	Trp	TGG	336	16.93	1.00
Y	Tyr	TAT	570	28.72	1.63
Y	Tyr	TAC	128	6.45	0.37

### Phylogenetic analysis

**Figure 3:**
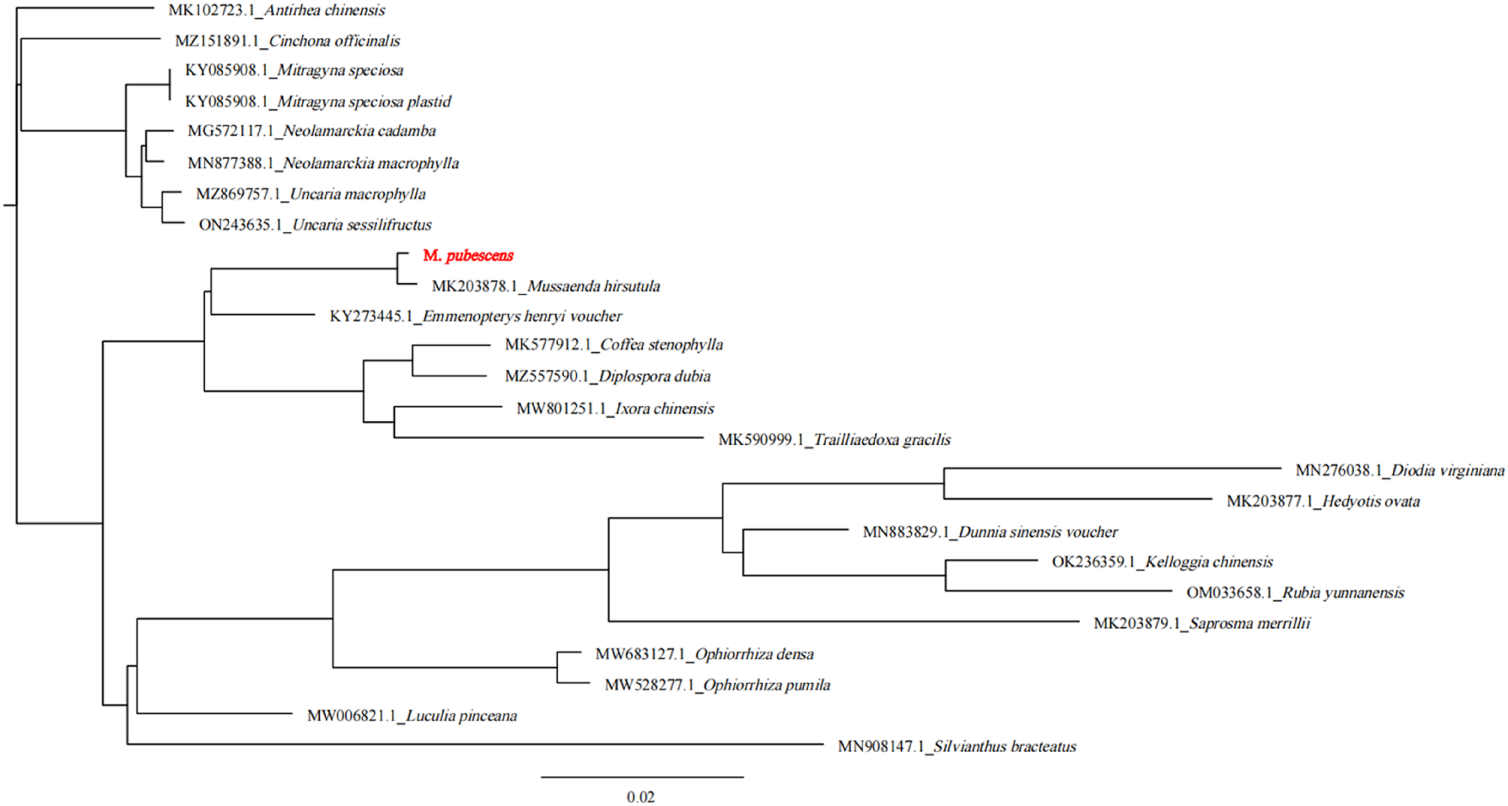
The phylogenetic tree of *M. pubescens.*

Chloroplast genomes are valuable sources of information for species identification and evolutionary analysis^19^. They serve as organelle-based “barcodes” to distinguish species and reveal interspecies phylogenetic relationships^43^. Furthermore, the progressive advancements in next-generation sequencing technology, specifically the implementation of second-generation technology, have facilitated the simplification of chloroplast genome sequencing. Thus, an accumulating body of studies has employed complete chloroplast genome sequences to examine phylogenetic relationships within angiosperms. The phylogenetic position of *M. pubescens* was analyzed by downloading 19 complete cp genomes from the GenBank database, all of which belonged to the *Gramineae* family. The genomes were aligned using the MAFFT^24^, and the phylogenetic tree was constructed with the Mega-X v10.0.5 software^44^ employing the maximum likelihood method and 1000 bootstrap replicates (Fig. 3). The observed cp genome sequences played a vital role in elucidating and comprehending the phylogenetic relationships among *Mussaenda* species.

The diversification of the species, *M. pubescens*, was attributed to the presence of a highly diverse set of genes, intermolecular recombination in the LSC region, and the co-occurrence of tandem repeats. The phylogenetic analyses yielded highly similar topologies across the complete cp genomes, LSC regions, and SSC regions (the complete cp genome displayed complete consistency with the LSC region), and every node in the phylogenetic trees exhibited high bootstrap support, with the exception of *Antirhea chinensis* and *Cinchona officinalis* (Fig. 3). *Rubiaceae* species, along with species in the same genus, showed a tendency to cluster together on a single large branch. The *Rubiaceae* branch was divided into two clades with *Antirhea*, *Cinchona*, *Mitragyna*, *Neolamarckia*, and *Uncaria* to other 20 genera. *Mussaenda* was found to be related to 19 other genera, and within these, it was determined that *Mussaenda pubescens* shared a close relationship with five other genera (*Mussaenda hirsutula*, *Emmenopterys*, *Coffea*, *Diplospora*, *Lxora*, and *Trailliaedoxa*). The phylogenetic tree demonstrated that *Mussaenda* was polyphyletic and originated from the intercalation of branches from the genera *M. hirsutula*. Moreover, *M. pubescens* and *M. hirsutula* were found to be closely related genera. The findings support the identification and taxonomic study of *M. pubescens*, enabling resource collection, cultivation, study of pharmacological activities, and development of functional products.

## Conclusion

*M. pubescens* was compared with the specimens of Chinese *Mussaenda*, indicating that *M. pubescens* was closely related to *M. hirsutula*. However, *M. pubescens* was distinguished by its gyro-shaped corolla tubes measuring 3–4 mm in length, ovate oblong or ovate lanceolate leaves, and oblong lanceolate corolla lobes. Furthermore, this study determined the complete cp genomes of *M. pubescens* for the first time and revealed their basic structures, conservation, and variability. The complete cp genomes, including their LSC and SSC regions, were employed to investigate the robust phylogenetic relationships within and between genera. Additionally, the availability of this information would offer useful references for subsequent studies on taxonomic identification, phylogenetics, population structure, and biodiversity within the genus *Mussaenda*. Furthermore, the comparative analysis of cp genomics in the genus *Mussaenda* contributed to our comprehension of cp genome dynamics, complexity, and evolution within the *Rubiaceae* family. In conclusion, this study served as a valuable reference for future research on species identification, evolutionary relationships, and the development of genetic resources within the *Rubiaceae* family.

## Data Availability

The complete chloroplast genome has been submitted to the Database Resources of the National Genomics Data Center, China National Center for Bioinformatio (Genome Sequence Archive accession number: CRA013306; https://ngdc.cncb.ac.cn/gsa).
